# Severe measles with pneumonitis in an immunocompetent adult

**DOI:** 10.1016/j.clinme.2024.100239

**Published:** 2024-08-30

**Authors:** Danielle Lee, Oliver Mercer, Varsha Halai, Laura Gill, Colin Macleod, Temi Lampejo

**Affiliations:** King's College Hospital NHS Foundation Trust, London, United Kingdom

**Keywords:** Measles, Pneumonitis, Immunocompetent, Ribavirin

## Abstract

Measles is a highly contagious but vaccine-preventable airborne-transmitted viral infection of which there has been a recent resurgence of cases worldwide over the past year, including in countries such as the UK, which had previously successfully achieved endemic measles elimination through vaccination programmes. Measles is typically a self-limiting illness, but can rarely cause severe, life-threatening disease, particularly when complicated by respiratory or neurological involvement. These severe complications are not typically seen in the absence of immunosuppression. We describe a rare case of severe measles with pneumonitis in an immunocompetent adult necessitating admission to an intensive care unit (ICU).

## Case presentation

A 44-year-old man presented to our hospital emergency department with a 1-week history of malaise, myalgia, dry cough and diarrhoea, followed by a 1-day history of fever and a widespread rash. He had recently been in contact with his two grandchildren, who had both had confirmed measles 2 weeks earlier. He had no history of recent travel abroad and reported receiving all recommended childhood vaccinations in the UK, including measles vaccination. He was otherwise healthy with no past medical history, or any known underlying immunosuppression.

**Fig. 1:**
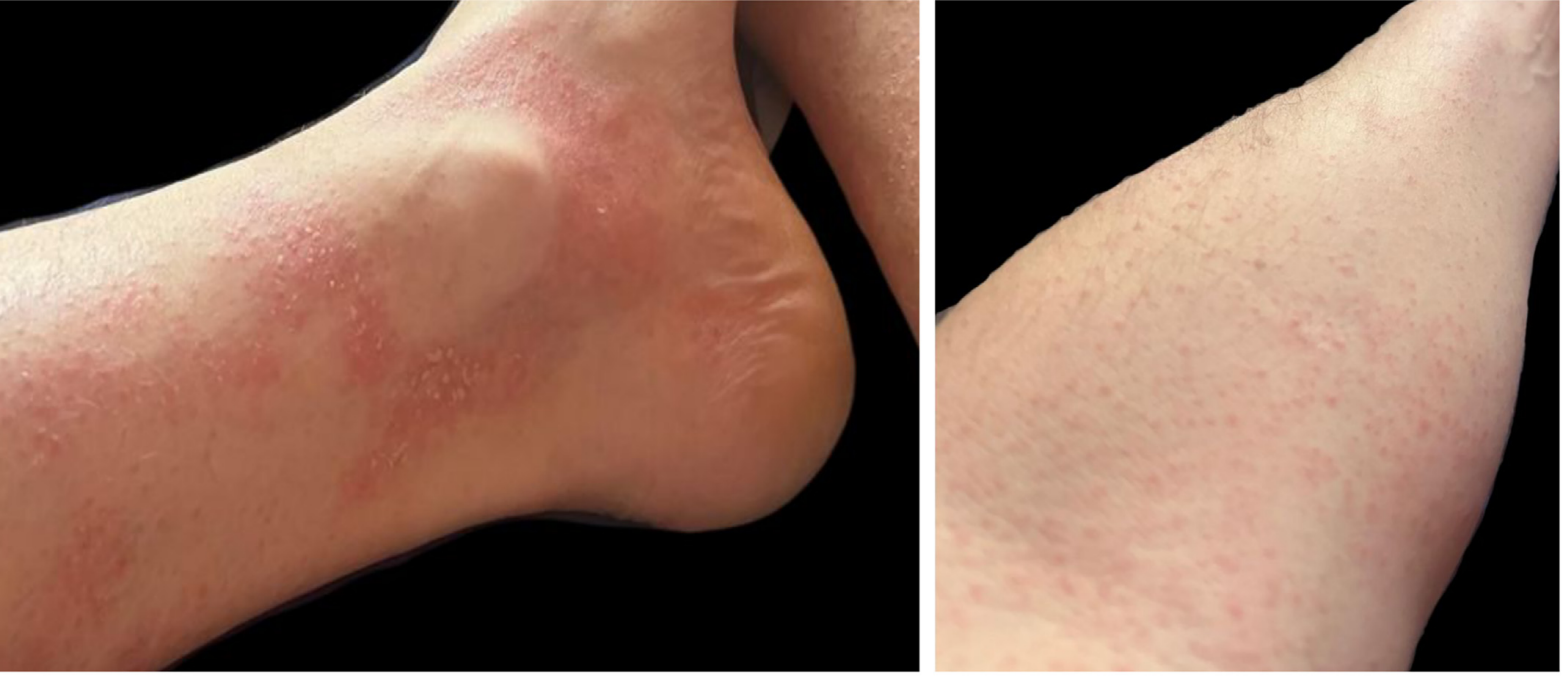
Photographs taken by the patient demonstrating a characteristic measles morbilliform rash seen on the left forearm and lower limbs.

**Fig. 2:**
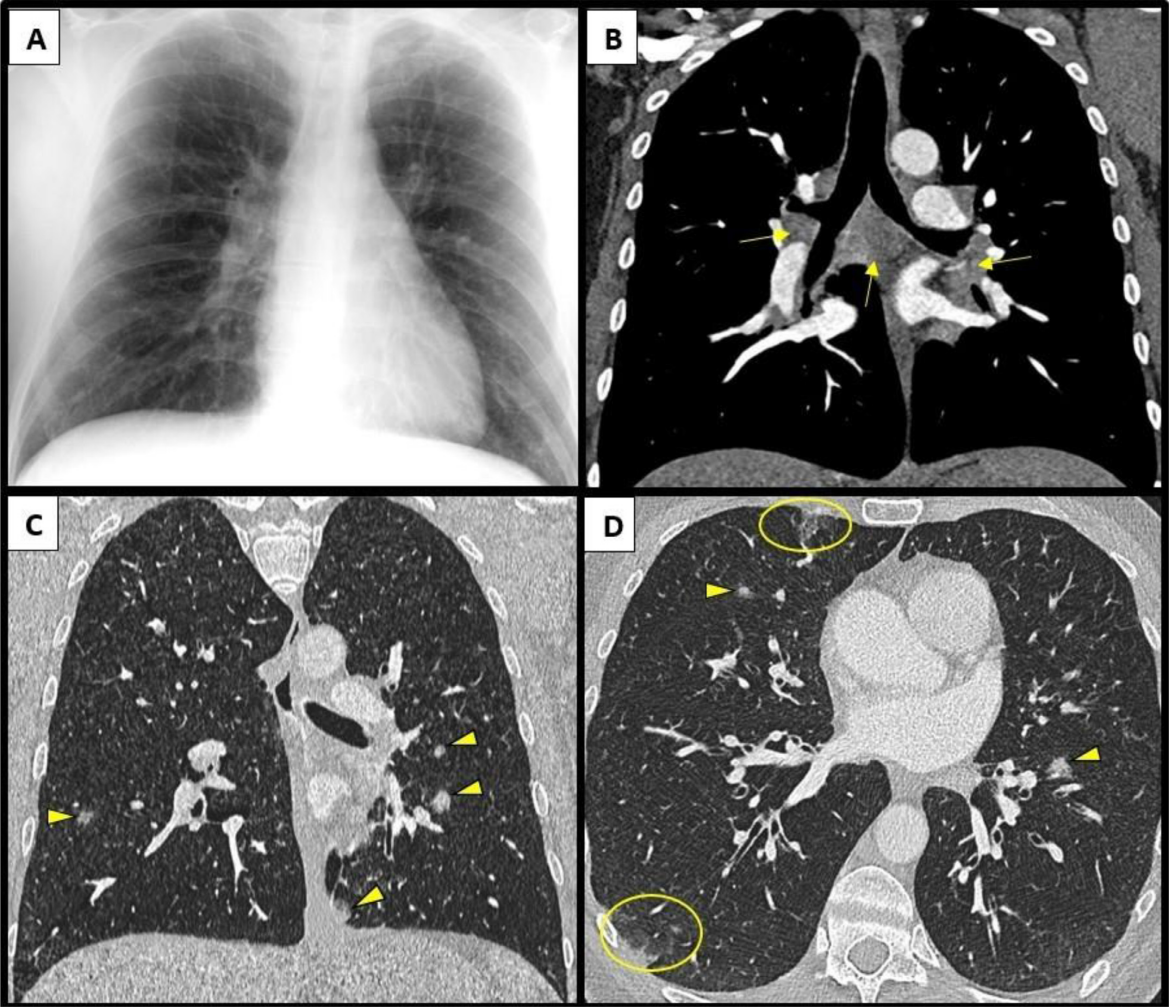
Chest radiograph and computed tomography images of the thorax. (A) Chest radiograph demonstrates faint multifocal nodular opacities within both lower zones and a bulky right hilum. (B) Coronal computed tomography image of the thorax in a soft tissue window confirms the suspected right hilar adenopathy and shows further adenopathy at the left hilum and within the mediastinum (arrows). (C) Coronal and (D) axial computed tomography images of the thorax in lung windows show multifocal nodular consolidation (arrow heads) and focal ground glass opacities (circled). Bibasal bronchial wall thickening is also seen in the axial images.

On examination, he was pyrexial with a widespread blanching erythematous maculopapular rash (Fig. 1). No Koplik spots were seen. He was hypoxic, requiring 3 L supplementary oxygen via nasal cannulae, and tachypnoeic at 25–30 breaths per minute, therefore requiring hospital admission. Blood tests showed raised inflammatory markers (C-reactive protein 36 mg/L (normal <5 mg/L)) and lymphopenia (lymphocytes 0.69 × 10^9^/L (normal 1–4 × 10^9^/L)). Serum liver function tests showed a raised aspartate aminotransferase 147 U/L (normal 10–50 U/L) and gamma-glutamyl transferase 148 U/L (normal 1–55 U/L), with normal alkaline phosphatase 98 U/L (normal 30–130 U/L) and bilirubin 8 U/L (normal 1–55 U/L). He had a mild thrombocytopenia (platelets 143 × 10^9^/L (normal 150–450 × 10^9^/L)) but otherwise normal full blood count, coagulation, fibrinogen and renal function tests. HIV serology was negative. A chest radiograph demonstrated multifocal nodular opacities within both lower zones and right hilar adenopathy, suggesting infective/inflammatory aetiology (Fig. 2A). He was admitted to a single room with airborne precautions and notified to the local health protection team as a suspected case of measles. A mouth swab subsequently tested positive for measles virus RNA, confirming the diagnosis.

He rapidly deteriorated on the day of admission, with progressively worsening hypoxia and breathlessness requiring ICU admission for high-flow-nasal oxygen (HFNO); FiO_2_ 60 % at a flow rate of 60 L/min to maintain oxygen saturations of 94–98 %. A computed tomography pulmonary angiogram was negative for pulmonary embolism, but showed widespread inflammatory change with multifocal nodular consolidation and ground glass opacities, bibasal bronchial wall thickening and reactive bihilar and mediastinal adenopathy (Fig. 2B-D). He was commenced on oral ribavirin (400 mg twice daily) for measles pneumonitis and his antibiotics were escalated from doxycycline to levofloxacin (he reported a penicillin allergy).

He improved clinically over the next few days and was weaned off supplementary oxygen. He tolerated the ribavirin well with no evidence of haemolytic anaemia (ribavirin's primary dose-limiting side effect). He completed 4 days of ribavirin and antibiotics after which he was discharged home, making a full recovery. He remained well and was asymptomatic at outpatient review 1 month later.

## Discussion

Measles is a highly contagious airborne-transmitted viral infection caused by the measles virus, a member of the genus *Morbillivirus* and family Paramyxoviridae.^1^ Our patient had a clear measles exposure history but, given its highly transmissible nature, many patients will not be aware of a prior exposure. It typically presents with a 2–4-day prodrome of fever, malaise, cough, coryza and conjunctivitis followed by a characteristic generalised erythematous maculopapular rash, usually starting on the face and then spreading to the trunk and extremities. Koplik spots (present in 40–70 % of cases) may be seen 48 hours prior to rash onset, which are characterised by clusters of white/grey lesions on the buccal mucosa and are considered to be pathognomonic for measles. Measles has an incubation period of 7–21 days and individuals are generally considered infectious from 4 days before until 4 days after rash onset.^1^

There has continued to be a concerning rise in measles cases globally over the past year, including in higher-income settings such as the UK, which had previously achieved endemic measles elimination, and is predicted to lead to further significant measles outbreaks in more than half the countries of the world in 2024.^2^ The UK experienced a large outbreak in Birmingham in late 2023, with rising cases being reported in London and other UK regions in recent months.^3^ This is felt to have been driven by a combination of factors, including vaccine hesitancy with reduced vaccine uptake during and after the COVID-19 pandemic, and a lack of awareness of the potentially serious consequences of measles.^2^ Given his history of receiving measles vaccination as a child, it is particularly surprising that our patient developed severe measles pneumonitis. One dose of a measles-containing vaccine is considered to have a protective efficacy of at least 95 %, increasing further following a second dose.^4^ It is possible that our patient's vaccination history was misremembered, although reports of breakthrough measles infection exist in previously vaccinated individuals due to waning immunity.^5^

Although most measles cases are self-limiting, very rarely severe, potentially fatal complications with multi-system involvement can occur. The risk of complications from measles is increased in children younger than 5 years, pregnant women and individuals who are immunocompromised or malnourished.^1^ Respiratory complications including primary measles pneumonitis are rare, but are associated with the highest measles-associated morbidity and mortality.^6^ Our case is very unusual given that there was no evidence of immunocompromise, although our patient did have a heavy smoking history that may have increased his risk of respiratory involvement. Neurological disease presenting as encephalitis (in its various measles-associated forms) is another even rarer severe complication.^1^ Our patient had no evidence of neurological involvement.

The management of measles is predominantly supportive, given the self-limiting nature, and there are currently no approved antivirals. However, some evidence exists that ribavirin may help to reduce duration of symptoms and hospitalisation, and the development and severity of measles-related complications, especially respiratory complications such as pneumonitis.^7^ Conclusive data supporting the use of ribavirin in severe measles are limited and therefore guidelines for its use are lacking.^8^ We used ribavirin for our patient in view of his life-threatening disease and he received it orally as this was the form readily available at our trust, although it can also be given via a nebuliser or intravenously. Given that he was receiving ribavirin for an unusual and unlicensed indication with no specific guidelines, we cautiously opted for the lowest dose of ribavirin typically used in adults (400 mg twice daily) to minimise the risk of haematological toxicity. However, there was scope to give a higher dose (15–20 mg/kg/day in two divided doses) as has been used for the treatment of other infections such as respiratory syncytial virus (RSV). Use of ribavirin in any form is contraindicated in pregnant women due to its teratogenicity,^9^ and barrier contraception should generally be used for 6 months following use in women and men of childbearing age.

Another treatment that has been used for severe measles is vitamin A therapy, although the evidence for its use in adults is extremely limited.^7^ Vitamin A deficiency is associated with more severe disease and complications from measles, and treatment with vitamin A supplementation is therefore recommended by the World Health Organization (WHO) for children under 5 years old with suspected measles.^8^ The added benefit of vitamin A therapy in measles is more evident in resource-limited settings where vitamin A deficiency may be more prevalent.

## Conclusion

This case emphasises the importance of increasing awareness of measles among healthcare professionals, particularly in light of recent outbreaks, so as to facilitate prompt recognition, testing, isolation and reporting of suspected cases. It also highlights that severe life-threatening complications of measles can rarely occur in otherwise healthy adults. Although evidence supporting the use of ribavirin is limited, early use may be beneficial in individuals with measles pneumonitis necessitating hospital admission, particularly if immunocompromised and/or with underlying pulmonary disease.

## Funding

Not applicable.

## Patient consent

Written consent was obtained from the patient to publish.

## Ethical statement

Ethical approval was not deemed to be required for this case report.

## CRediT authorship contribution statement

**Danielle Lee:** Writing – review & editing. **Oliver Mercer:** Writing – review & editing. **Varsha Halai:** Writing – review & editing. **Laura Gill:** Writing – review & editing. **Colin Macleod:** Writing – review & editing. **Temi Lampejo:** Conceptualization, Writing – review & editing.

## Declaration of competing interest

The authors declare that they have no known competing financial interests or personal relationships that could have appeared to influence the work reported in this paper.
